# Social buffering of the stress response: insights from fishes

**DOI:** 10.1098/rsbl.2022.0332

**Published:** 2022-10-26

**Authors:** Kathleen M. Gilmour, Brittany Bard

**Affiliations:** Department of Biology, University of Ottawa, 30 Marie Curie, Ottawa, ON, Canada K1N 6N5

**Keywords:** cortisol, hypothalamic–pituitary–interrenal axis, social context, behaviour, isotocin

## Abstract

Social buffering of stress refers to the effect of a social partner in reducing the cortisol or corticosterone response to a stressor. It has been well studied in mammals, particularly those that form pair bonds. Recent studies on fishes suggest that social buffering of stress also occurs in solitary species, gregarious species that form loose aggregations and species with well-defined social structures and bonds. The diversity of social contexts in which stress buffering has been observed in fishes holds promise to shed light on the evolution of this phenomenon among vertebrates. Equally, the relative simplicity of the fish brain is advantageous for identifying the neural mechanisms responsible for social buffering. In particular, fishes have a relatively small and simple forebrain but the brain regions that are key to social buffering, including the social behaviour network, the amygdala and the hypothalamic–pituitary–adrenal/interrenal axis, are functionally conserved across vertebrates. Thus, we suggest that insight into the mechanistic and evolutionary underpinnings of stress buffering in vertebrates can be gained from the study of social buffering of stress in fishes.

## 1. Introduction

Research across a range of vertebrate animals indicates that social interactions can influence the behavioural, neural and endocrine responses to environmental challenges (e.g. [1–4]). Whether the effects are positive or negative depends on the nature of the social interactions as well as the social environment of the animal. For example, the formation of social dominance hierarchies can result in subordinate individuals experiencing chronic stress as indicated by persistent elevation of glucocorticoid stress hormones [5,6]. By contrast, the presence of a conspecific can reduce the corticosterone response to a stressor in rodents (e.g. [7,8]), an effect of social interaction commonly termed ‘social buffering’ (reviewed by [9–12]). Social buffering is viewed as beneficial because it lowers stress hormone levels and promotes sociality [9]. Although the negative effects of social interactions on stress responses have received considerable attention [13], few studies have examined social buffering of stress in non-mammalian vertebrates. Here, we review recent findings on the social buffering of stress in fishes, highlighting the potential usefulness of fish, as early branching vertebrates, for providing insight into the evolution of social buffering as well as in uncovering the mechanisms underlying this phenomenon.

In mammalian studies, the term ‘social buffering’ is limited typically to social relationships among conspecifics that are affiliative in nature. The individuals need not be familiar with each another, but must not engage in agonistic interactions [9]. Thus, the blunted corticosterone response to restraint stress in subordinate rats that have elevated baseline corticosterone levels because of chronic social stress [14] does not constitute social buffering. Equally, then, comparable scenarios in fishes should not be considered social buffering, such as the blunted cortisol response to acute stress observed in subordinate salmonid fishes experiencing elevated baseline cortisol levels caused by chronic social stress [15,16]. Classically, the endpoints measured in mammals for the identification of social buffering have been those associated with the stress response. When the mammalian hypothalamic–pituitary–adrenal (HPA) axis is activated, neurons in the paraventricular nucleus (PVN) of the hypothalamus secrete corticotropin-releasing hormone (CRH), which acts on the corticotropes of the anterior pituitary to stimulate the secretion of adrenocorticotropic hormone (ACTH). In turn, ACTH stimulates glucocorticoid production by the adrenal cortex [12]. Neuroendocrine stress responses are strongly conserved across vertebrates [17,18]. Thus, the indicators of social buffering used in mammals—changes in HPA axis activity, as well as accompanying changes in behaviour and/or changes in the central nervous system that alter HPA axis activity [9]—may also be used in fishes.

## 2. Does social buffering of stress occur in fishes?

Empirical evidence indicates that social buffering of stress occurs in fishes. For example, three-spined stickleback (*Gasterosteus aculeatus*) individually exposed to a simulated predator exhibited higher cortisol levels than fish that experienced the apparent predation risk as a group [19]. Similar results were obtained for cortisol levels in zebrafish (*Danio rerio*) that were exposed to the stressor of a novel environment either alone or in groups [20]. Also, zebrafish exposed to a stressor when in the presence of conspecifics exhibited less ‘freezing’ than did fish that were alone; ‘freezing’ is a stereotyped behavioural response to a threat [21]. Faustino *et al*. [21] placed the conspecifics in a separate tank, eliminating physical contact with the test fish, and examined the importance of visual versus olfactory cues in permitting social buffering. Although either cue was sufficient to induce social buffering, the effect persisted for longer when the test fish had sight of the conspecifics, making it the more effective sensory cue. Stickleback and zebrafish are shoaling species, forming social groups largely lacking in hierarchical structure, although aggressive interactions may occur during breeding events [22,23]. At least in zebrafish, even shoals as small as two fish were effective in permitting social buffering [21]. Juvenile lake sturgeon (*Acipenser fulvescens*) also form loose aggregations, in this case with no evidence of agonistic interactions or social hierarchy formation [24]. Here again, a comparison of cortisol levels following a stressor consisting of a rapid increase in water temperature supported the occurrence of social buffering, with the rise in cortisol being higher and the recovery to baseline levels slower in single fish than in fish exposed to the stressor in groups [25]. A species with a more elaborate social structure is the daffodil cichlid (*Neolamprologus pulcher*), a cooperative breeder that lives in relatively stable, size-structured social groups consisting of a dominant breeding pair with (smaller) subordinate helpers [26,27]. Group members regularly engage in affiliative behaviours, recognize and prefer group members and share the work of territory defence and brood care. Cortisol levels in subordinate daffodil cichlids held alone following a netting stressor were higher than those in fish that recovered within their social group [28]. Interestingly, this social buffering occurred even though the test fish received fewer acts of affiliation from its group members after the stressor than before; in fact, there was a transient increase in aggression toward the test fish immediately following the stressor [28].

The examples above focused on species that largely live in groups, although with different levels of social organization. By contrast, the weakly electric brown ghost knifefish (*Apteronotus leptorhynchus*) is a more solitary species that is active at night and seeks shelter during the day, with male fish, in particular, preferring to be alone in their shelter [29,30]. In this species, stress-induced inhibition of brain cell proliferation was attenuated in individuals that shared a tank with a conspecific [31]. Tail injury mimicking a predator attack reduced cell proliferation in the forebrain, and this effect was mitigated by the presence of a tank mate before and/or after the injury, i.e. addition of an unfamiliar conspecific after the injury was sufficient to provide social buffering [31]. Tank mates were separated by a mesh divider that permitted visual, chemical and electrical contact while preventing physical contact [31]. Social interaction is similarly known to mitigate stress-induced reductions in neurogenesis in mammals [32].

Countering these examples where stress responses were tempered by social buffering are a few studies in which the presence of conspecifics failed to lower stressor-induced cortisol levels. For example, group-housed zebrafish exhibited higher cortisol levels than socially isolated zebrafish in response to a novel environment, a social stimulus or a chasing stressor, although not a predator [33–35]. In addition, no difference in stressor-induced cortisol levels was detected between group-held and socially isolated African catfish (*Clarias gariepinus*) [36]. It should be noted that in these studies socially isolated fish were held on their own for periods of 15 days to 6 months, whereas zebrafish were separated from their group no more than 24 h prior to stressor exposure in the studies that observed social buffering [20,21]. Although the effects of social isolation on baseline cortisol levels in zebrafish remain equivocal [33,35,37,38], the potential for prolonged social isolation to alter stress axis function complicates interpretation of stress responses and therefore the detection of social buffering [11]. Using pumpkinseed sunfish (*Lepomis gibbosus*) angled from a lake and sampled post-capture on the research vessel, Belanger *et al*. [39] found similar cortisol responses to capture stress in fish held singly or in groups. The absence of social buffering, in this case, may reflect the constraints of field conditions, including lack of control over whether fish held in groups were from the same shoal. Alternatively, social buffering of stress may not occur in this species, or may only occur under environmental conditions that were not met in this field experiment. As in mammals [11], it is unlikely that social buffering occurs in all fish species.

**Figure 1.**
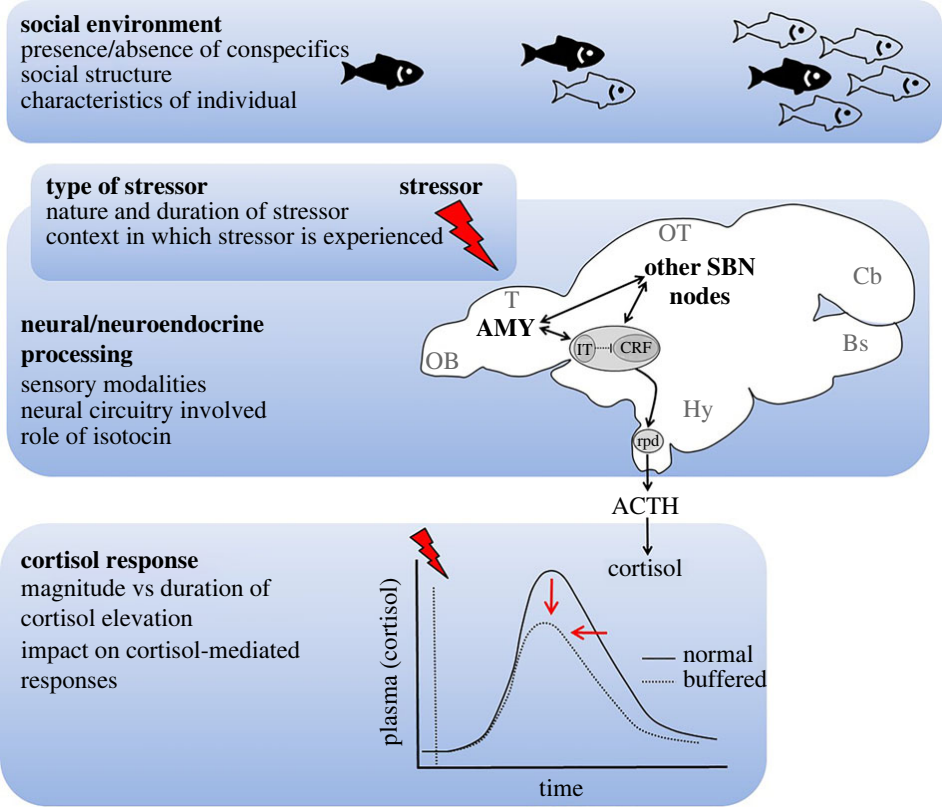
A summary figure that lists factors that may affect social buffering of stress in fishes. Social buffering may be affected by individual characteristics (e.g. stress-coping style, social status, physiological condition) and/or the type of social structure (e.g. pair-bonds, social cohesion, group size and stability). The duration (acute versus chronic) and nature (e.g. novel environment, predator, air exposure) of the stressor may influence social buffering. Likely candidates for the neural and neuroendocrine circuitry underlying social buffering include the piscine amygdala complex and the social behaviour network, and these must receive input from relevant sensory systems. Social buffering may be detected as reductions in the magnitude and/or duration of the cortisol response to a stressor, with downstream impacts on cortisol-mediated responses. *ACTH*, adrenocorticotropic hormone; *AMY*, amygdala; *Bs*, brainstem; *Cb*, cerebellum; *CRF*, corticotropic releasing factor; *Hy*, hypothalamus; *IT*, isotocin; *OB*, olfactory bulb; *OT*, optic tectum; *rpd*, rostral pars distalis, which contains the corticotropes; *SBN*, social behaviour network; *T*, telencephalon. The oval surrounding IT and CRF indicates the preoptic area (POA).

Collectively, then, the available evidence suggests that hormonal, behavioural and/or neural responses to a stressor can be tempered by social buffering in fishes. However, the small number of studies carried out to date limits the inferences and generalizations that can be drawn from the available data. Although studies have focused only on ray-finned fishes, social buffering has been reported in a basal actinopterygian (lake sturgeon) as well as in teleosts belonging to four different orders, suggesting a broad distribution. Social buffering in fishes does not appear to be limited to species that have highly structured societies or that form social bonds [40], but has also been observed in a solitary species (brown ghost knifefish) with an unfamiliar conspecific [31]. Visual and olfactory signals have been identified as sufficient sensory cues to indicate the presence of a social partner; tactile stimuli were not necessary, although sensory cues were examined in a single species [21] and are likely to vary according to the main sensory modalities of the species [9]. A variety of stressors has been used, but systematic comparisons among different types of stressors are needed, as is characterization of the dynamics of the cortisol response. Clearly, much remains to be learned about social buffering of stress in fishes (figure 1). Addressing these knowledge gaps will increase our understanding of stress physiology and social behaviour in fishes and may have practical applications for fish welfare considerations. There is, in addition, the potential to address questions about the evolution of social buffering and the neural mechanisms responsible for buffering effects.

## 3. Neural mechanisms of social buffering

Understanding of the neural circuitry underlying social buffering of stress remains limited even in mammals, likely at least in part because the neural mechanisms that are activated appear to differ depending on the nature of the social relationship [9,12]. There is evidence of both direct and indirect suppression of HPA axis activity during social buffering [9,10,12]. For example, mice exposed to the stressor of a novel environment had fewer c-Fos positive CRH neurons in the PVN if the exposure occurred with a conspecific or in the presence of bodily secretions of a conspecific. The immediate early gene c-Fos is used as an indicator of neuronal activity, and these results, therefore, support reduced activation of CRH neurons in a social buffering situation [41]. Subsequent work revealed that chemical compounds secreted by a conspecific activated a particular group of odorant receptors (the OR37 subfamily) [41]. The neuronal pathway that processes activation of OR37 is unusual in projecting to the PVN, and when mice were exposed to the stressor in the presence of OR37 ligands, a similar reduction in the number of c-Fos positive CRH neurons was observed [41]. These findings argue for a direct pathway through which olfactory cues from a conspecific can dampen activity of the HPA axis during exposure to a stressor. Activity of the HPA axis may also be modulated by the lateral amygdala and/or the prefrontal cortex. In rats, the presence of a conspecific during exposure to a fear-conditioned stimulus prevented HPA activation, and this response was associated with suppression of activity in the lateral amygdala [7,42,43]. Increased c-Fos induction in the prefrontal cortex together with a reduced cortisol response were detected in young guinea pigs exposed to the stressor of a novel enclosure in the presence of an unfamiliar adult male versus alone [44,45]. Supporting the possibility that this association was causal, the behavioural responses associated with social buffering in mice were elicited in the absence of a conspecific by optogenetic activation of a group of prefrontal cortex neurons, specifically those that had been active during previous social buffering by a conspecific [46].

Collectively, the examples above identify both direct and indirect neural circuits that appear to contribute to social buffering of stress in mammals. Whether comparable pathways underlie social buffering in fishes remains to be determined. The fish homologue of the mammalian PVN is the preoptic area (POA); POA neurons project to the anterior pituitary, releasing corticotropin-releasing factor (CRF) in the vicinity of the corticotropes that secrete ACTH [47]. Recently, a series of nuclei that forms a structure corresponding to the mammalian amygdala was identified in the forebrain of the zebrafish [48], and in addition, the fish brain contains the elements of the ‘social behaviour network’, a conserved collection of brain nuclei that regulates social behaviours across vertebrates [49,50]. However, to date only a single study has investigated activation of neural circuitry during social buffering in a fish. Faustino *et al*. [21] reported that there were no differences in the magnitude of neuronal activation (measured as *c-fos* transcript abundance), but that patterns of co-activation across various forebrain regions differed between zebrafish exposed to a stressor alone versus in the presence of conspecifics, suggesting that social buffering was associated with a specific pattern of activation of brain regions.

The neuropeptide oxytocin has been of widespread interest as a mediator of social buffering effects in mammals [9–12], largely owing to its well-established roles in the regulation of social behaviour and in the suppression of stress responses [51–55]. The most compelling evidence that oxytocin is involved in social buffering of stress has been obtained in prairie voles [56]. In female prairie voles recovering from a stressor, the social buffering effect provided by the presence of the male partner was blocked when the female was treated with an oxytocin receptor antagonist [57]. Equally, a social buffering effect could be elicited in females recovering alone by treating them with oxytocin [57]. Oxytocin levels in the PVN increased during exposure to the stressor but remained elevated during recovery only in females that recovered with their male partner [57]. Oxytocin-expressing neurons of the PVN project to the CRH-expressing neurons of the PVN as well as to brain regions that regulate the CRH-expressing neurons of the PVN [51,52,55]. Stress-induced oxytocin release appears to dampen the glucocorticoid stress response through direct and indirect effects on the CRF system as well as the HPA axis [51,52,55]. Oxytocin may also buffer stress by having effects that serve to reduce anxiety; the oxytocin-expressing neurons of the PVN project to various regions of the forebrain, and oxytocin-expressing neurons are found in a variety of brain regions [51,52,55].

By comparison, much less is known about isotocin, the fish homologue of oxytocin, in stress responses or regulation of the stress axis, although it is well-recognized as being involved in social behaviour in fishes [49,58]. Corresponding to the situation in mammals, isotocin is expressed in neurons of the POA that project widely throughout the brain, including to the forebrain and the hypothalamus [59–61]. Isotocin-expressing neurons also seem to project to the corticotropes of the anterior pituitary, at least in some species [62]. Also as in mammals, isotocin stimulates ACTH release from *in vitro* pituitary preparations [63,64], and there is evidence of increases in brain isotocin content in response to stressors such as high stocking density [65,66]. These observations suggest that isotocin may contribute to the regulation of stress axis activity in fishes through mechanisms similar to those in mammals. In addition, two somewhat equivocal observations hint at a potential role for isotocin in the social buffering of stress. Differences in transcript abundance of isotocin in the POA were detected between individuals of the cooperative breeder *N. pulcher* that were recovering from a stressor on their own versus with their social group. Somewhat unexpectedly, however, it was the individuals that recovered alone, and that had higher cortisol levels as a consequence, that exhibited higher transcript abundance of isotocin [28]. In zebrafish, higher whole-head isotocin levels accompanied by lower trunk cortisol levels were measured in individuals that recovered from a stressor with a group of familiar conspecifics versus those that recovered alone or with a group of unfamiliar conspecifics [67]. These results are intriguing, although it is challenging to interpret whole-head isotocin levels and the data only appear to be available in a pre-print repository. The sparsity of data in fishes on the potential role of isotocin and the neural circuitry activated during stress buffering serves to emphasize the need for additional work focused on determining the mechanisms that mediate social buffering of stress in fishes (figure 1).

## 4. Why study social buffering of stress in fishes?

Most studies of the social buffering of stress have focused on mammals, particularly species that are highly social and that form social bonds [9]. Among these species, the presence of a social bond between two individuals is the best predictor of whether social buffering will occur, although examples are known where social buffering is provided by the presence of an unfamiliar conspecific [9]. Viewed through this lens, social buffering may serve to enhance sociality, perhaps because gregariousness and social cohesion are promoted and reinforced by the ability of social partners to reduce a stress response [9,11]. Dampening of HPA axis activity through social buffering also may be important for group-living animals to reduce the negative effects associated with chronic activation of the HPA axis. Group living can result in social stress, particularly for individuals who occupy subordinate positions, and social buffering may offset this potential cost [1,68].

By contrast, the studies of social buffering of stress carried out to date in fishes have largely focused on species that are not considered to be strongly social or to form social bonds (see above, §2). The occurrence of social buffering in these species demonstrates that social bonds and sociality are not required for social buffering of stress. What, then, is the benefit of social buffering? Although sustained elevation of glucocorticoids is widely accepted to be damaging, a robust stress response is considered to be adaptive in coping with environmental challenges in the short term [69]. Thus, the value of blunting the cortisol response to an acute stressor is not obvious.

One possibility is that social buffering serves as a check on the social transmission of stress [70]. Social transmission of stress occurs when an individual animal that has not been exposed to a stressor exhibits behavioural and physiological stress responses through contact (e.g. visual, chemical) with a stressed conspecific. Although most studies have focused on mammals, primarily humans [70], there is also evidence of social transmission of stress in fishes [33,71–73]. For example, visual exposure to a predatory fish evoked a cortisol response in zebrafish that viewed the predator, but also in zebrafish that did not view the predator but observed only the zebrafish that were exposed to the predator [71]. Social transmission of stress may be useful, for instance in signalling the existence of a threat in the environment, but also has the potential to be maladaptive, if it leads, for example, to inappropriate amplification or prolongation of a stress response. By countering social transmission, social buffering may serve to avoid such maladaptive situations [70].

A second possibility is that social buffering enhances the capacity of individuals to withstand environmental challenges. For example *N*. *pulcher* tolerated lower water oxygen levels with a conspecific than while alone [74], and both three-spine stickleback [75] and mangrove rivulus (*Kryptolebias marmoratus*) [76], tolerated water temperatures that were further from their preferred values when a social group or conspecific was perceived to be present. Although plasma cortisol levels were not measured in these studies, it is tempting to speculate that lowering cortisol levels by social buffering contributed to the greater tolerance of fish exposed to the environmental challenge in the presence of a conspecific. In this regard, it is noteworthy that thermal tolerance was reduced in rainbow trout (*Oncorhynchus mykiss*) that experienced chronic elevation of plasma cortisol [77].

Ultimately, the benefit of social buffering of stress may lie in its contribution to endocrine flexibility, that is, in allowing the glucocorticoid response to a stressor to be modulated based on the individual's circumstances. The stress response plays a key role in adjusting an animal's behaviour and physiology, particularly its energy metabolism, to cope with environmental challenges. Increasingly there is recognition of flexibility in the endocrine stress response, i.e. individual variation in baseline and stress-induced hormone concentrations, in the magnitude or scope of the glucocorticoid response, and in the speed with which hormone levels increase and decrease [78–82]. The mechanisms responsible for such flexibility and its adaptive significance largely remain to be determined. By modulating stress hormone levels based on social context, social buffering of stress may serve as a mechanism of endocrine flexibility.

## 5. Concluding remarks

Our review of the literature clearly reveals that fishes have been understudied to date with respect to social buffering of the stress response. Yet, as the earliest branching vertebrates, they have much to offer to improve our understanding of the function of social buffering, as well as its evolution and relationship to sociality, and the physiological mechanisms through which it occurs (figure 1). Despite the relatively small and simple forebrain of fishes, there appears to be functional conservation across vertebrates of key brain regions, including the social behaviour network, the amygdala, and the HPA (HP-interrenal in fishes) axis [17,18,48–50]. Given this conservation, the relative simplicity of the fish brain is likely to be advantageous in identifying the neural mechanisms responsible for social buffering. A second advantage of studying social buffering in fishes is the breadth of social behaviour and social systems that exists across group-living fishes (even among related species), which will provide the diversity that is needed to dissect relationships between social buffering and sociality. In short, we suggest that insight into the mechanistic and evolutionary underpinnings of social buffering of stress in vertebrates can be gained from the study of social buffering in fishes.

## Data Availability

This article has no additional data.
